# Therapeutical Novelties

**Published:** 1854-11

**Authors:** 


*Therapeutical Novelties.*—Mr. A. I. Mathews, druggist, of this city, has sent us a specimen package of Dr. Clertan's "Perles d' Ether," a new preparation now in use in Paris. The ether pearls are simply four or five drops of pure ether inclosed in a capsule, which readily dissolves in the fluids of the stomach. The pearls are as large as a large pea, and are taken as pills. Their advantage is found in the accuracy of the dose, the freedom from unpleasant taste or odor, and the avoidance of all loss by evaporation. The volatile nature of ether renders its neat administration a somewhat difficult feat, and its effect consequently somewhat uncertain. All these objections seem to be overcome in the "Perles d' Ether."

Accompanying the above was a copy of Lindsay & Blakiston's Physicians' Visiting List for 1855, which may be found hereafter at Mathews. Its merits need no comment. Mr. Mathews has also a very clever demulcent, called Acacia Paste, of which Gum Acacia, Marsh Mallows, and Iceland Moss, are the ingredients. It is a good and reliable substitute for those cough mixtures containing opiates, which are doing so much mischief. H.

